# Coexisting social conditions and health problems among clients seeking treatment for illicit drug use in Finland: The HUUTI study

**DOI:** 10.1186/1471-2458-13-380

**Published:** 2013-04-23

**Authors:** Ifeoma N Onyeka, Caryl M Beynon, Hanna Uosukainen, Maarit Jaana Korhonen, Jenni Ilomäki, J Simon Bell, Mika Paasolainen, Niko Tasa, Jari Tiihonen, Jussi Kauhanen

**Affiliations:** 1 Institute of Public Health and Clinical Nutrition, Faculty of Health Sciences, University of Eastern Finland, P.O.Box 1627, Kuopio, FI, 70211, Finland; 2 Centre for Public Health, Faculty of Health and Applied Social Sciences, Liverpool John Moores University, Liverpool, United Kingdom; 3 School of Pharmacy, Faculty of Health Sciences, University of Eastern Finland, Kuopio, Finland; 4 Department of Pharmacology, Drug Development and Therapeutics, University of Turku, Turku, Finland; 5 Sansom Institute, School of Pharmacy and Medical Sciences, University of South Australia, Adelaide, Australia; 6 Helsinki Deaconess Institute, Helsinki, Finland; 7 Department of Forensic Psychiatry, Niuvanniemi Hospital, University of Eastern Finland, Kuopio, Finland; 8 National Institute for Health and Welfare, Helsinki, Finland; 9 Department of Clinical Neuroscience, Karolinska Institute, Stockholm, Sweden

**Keywords:** HUUTI study, Illicit drug use, Homelessness, Depression, Hepatitis C, Comorbidity

## Abstract

**Background:**

Illicit drug use is an important public health problem. Identifying conditions that coexist with illicit drug use is necessary for planning health services. This study described the prevalence and factors associated with social and health problems among clients seeking treatment for illicit drug use.

**Methods:**

We carried out cross-sectional analyses of baseline data of 2526 clients who sought treatment for illicit drug use at Helsinki Deaconess Institute between 2001 and 2008. At the clients’ first visit, trained clinicians conducted face-to-face interviews using a structured questionnaire. Logistic regression was used to compute adjusted odds ratios (AORs) and 95% confidence intervals (CIs) for factors associated with social and health problems.

**Results:**

The mean age of the clients was 25 years, 21% (*n* = 519) were homeless, 54% (*n* = 1363) were unemployed and 7% (*n* = 183) had experienced threats of violence. Half of the clients (50%, *n* = 1258) were self-referred and 31% (*n* = 788) used opiates as their primary drugs of abuse. Hepatitis C (25%, *n* = 630) was more prevalent than other infectious diseases and depressive symptoms (59%, *n* = 1490) were the most prevalent psychological problems. Clients who were self-referred to treatment were most likely than others to report social problems (AOR = 1.86; 95% CI = 1.50–2.30) and psychological problems (AOR = 1.51; 95% CI **=** 1.23–1.85). Using opiates as primary drugs of abuse was the strongest factor associated with infectious diseases (AOR = 3.89; 95% CI = 1.32–11.46) and for reporting a combination of social and health problems (AOR = 3.24; 95% CI = 1.58–6.65).

**Conclusion:**

The existence of illicit drug use with other social and health problems could lead to increased utilisation and cost of healthcare services. Coexisting social and health problems may interfere with clients’ treatment response. Our findings support the call for integration of relevant social, medical and mental health support services within drug treatment programmes.

## Background

Illicit drug use has become a major concern in Europe, with high estimates of past year use of cannabis (23 million), cocaine (4 million), MDMA (‘ecstasy’, 2.6 million) and amphetamines (2 million) among 15 – 64 years olds [1]. Among the same age group in Nordic countries, lifetime prevalence of cannabis use is 36.5% in Denmark, 16.2% in Norway, 14.3% in Finland, and 12.5% in Sweden [2]. In response, many countries have initiated drug treatment programmes to counter negative health and social consequences of illicit drug use. In Finland, those seeking treatment for drug use have access to both specialised and generic services. Specialised services are provided through outpatient clinics, short-term inpatient care, rehabilitation units and support services. Treatment services are also available through primary, social and health care services [3].

Use of illicit drugs is associated with emotional and behavioural changes which make drug-taking assume precedence over other areas such as school, work, family and other relationships [4]. An American longitudinal education survey, initiated prior to high school entry, indicate that drug users have fewer years of schooling [5]; this might impact negatively on their career prospects and predispose them to social disadvantage due to difficulties in finding and maintaining jobs [6]. Due to poor financial situation, their living conditions deteriorate, stable housing becomes unaffordable and some may end up living on the streets [7,8]. Family responsibilities are also affected: for those who live with children, the risk of neglect/child abuse increases due to drug-related poor parenting skills [9].

Illicit drug use has also been implicated in the spread of blood-borne infectious diseases such as HIV and Hepatitis C due to sharing of non-sterile injecting equipment by injecting drug users (IDU) [10-12]. However, the risk of infectious diseases is not limited to IDUs. Transmission among non-injecting drug users has been reported to occur when a person infected with a blood-borne virus shares straw/tubing to snort drugs with an uninfected person, where they share other items which could convey blood such as toothbrushes, and through a sexual relationship with a sero-negative partner [13]. Through these routes, the risk of becoming infected is low compared to sharing infected needles/syringes. Irrespective of route of drug administration, the risk of blood-borne viruses being transmitted between drug users, and indeed between drug users and non-drug users through social and sexual interactions, should be considered an important public health issue. Other infections and health problems reported among drug users include abscesses, cellulitis, bacterial endorcarditis, necrotizing fasciitis, osteomyelitis, candidosis, thrombophlebitis, and ulcers [14].

The prevalence of psychological disorders is known to be high among those who use illicit drugs [15]. Such disorders may precede drug use [16] and they may also occur as a result of it [17]. Co-occurring psychological disorders have been found to lower clients’ readiness to change their addictive behaviours [18], resulting in poor treatment outcomes. Clients experiencing both drug addiction and psychological disorders tend to use more services, have higher relapse and re-admission rates thereby increasing the cost of treatment [19].

Identifying conditions that coexist with drug use is necessary for planning and provision of additional services based on identified needs. The epidemiological part of the “huumehoito tietokanta” (HUUTI, translated as drug treatment database) consortium research project is the first large-scale longitudinal study of illicit drug users seeking treatment in Finland. The aim of this paper is to describe the prevalence and factors associated with social and health problems that coexist with drug use based upon information provided by clients at their first visit to Helsinki Deaconess Institute (HDI).

## Methods

### Study design

The original sample consisted of a non-random sample of 4817 clients who sought treatment at HDI between 31 January 1997 and 31 August 2008. However, to minimize the amount of missing data for some variables of interest, this analysis was restricted to a subset of clients who attended the HDI between 2001 and 2008. There were no selection criteria: all consecutive clients who sought treatment at HDI during the study period were included in the study. This paper is based upon information provided by each of the clients during their first visit (i.e. their first face-to-face interview) at HDI, which serves as the baseline for subsequent longitudinal analyses.

### Subjects

A total of 2526 clients (1702 males and 824 females) sought treatment at the HDI between 2001 and 2008. The HDI is a large drug treatment provider in Helsinki and clients came from the Greater Helsinki area including some surrounding semi-rural communities. Treatment-seeking at HDI was based on client self-referral, referral from doctors or other treatment centres, and other sources. Following initial assessment during the first visit, treatment plans were drawn up and clients were assigned to various drug treatment modalities for subsequent visits. Ethical approvals were obtained from the Research Ethics Committee of the Hospital District of North-Savo and the Ethics Committee of HDI. Permissions were obtained from the Ministry of Social Affairs and Health of Finland and from appropriate municipal authorities of all Greater Helsinki area communities where clients resided.

### Instrument

Items on the questionnaire were adapted from the European Addiction Severity Index (EuropASI), the Treatment Demand Indicator Protocol (TDI) and other questions, which were relevant for evaluation of treatment needs and clinical monitoring of clients. The EuropASI is the European version of Addiction Severity Index (ASI) [20,21] which contains questions on drug use, employment/support, family/social history, psychiatric disorders, medical and legal issues. Researches have demonstrated the reliability and validity of the EuropASI [22,23]. The TDI, developed by the Pompidou Group of the Council of Europe [24-26], contains questions on treatment contact, sociodemographic information and drug use. The reliability of self-reported data using TDI was estimated to be 90% [24].

### Data collection

At first visit, trained clinicians interviewed clients using a structured questionnaire to obtain self-reported information on: drug use history, accommodation, employment and social history and medical and psychiatric histories. Clients provided information about their main reason for seeking treatment, referral to treatment, previous contact with any drug treatment centre, and whether they were receiving concurrent treatment elsewhere. To assess their social situations and demographics, clients responded to questions about their level of education; marital status; employment; and main source of income at the time of interview. They also responded to questions about their children under 18 years and other drug users living in the same household. Homelessness was defined as the presence or absence of postal code/address. Questions were asked about threats of violence from anybody (i.e. external sources). Infectious disease status was based on self-reports from clients based upon their perception of previous laboratory investigations. Clients were asked if they had been screened for infectious diseases (HIV, Hepatitis A, Hepatitis B and Hepatitis C) in the past and for the results of the tests. Data on depressive symptoms were based on self-reports. The clinicians (nurses and physicians who specialised in psychiatry and addiction medicine) asked further questions to assess other mental health conditions including psychosis and suicidal thoughts and/or attempts.

### Data analysis

Variables were expressed as a proportion of the denominator (2526). Factors associated with social problems and heath problems (infectious diseases and psychological problems) were initially assessed in univariate logistic regression models. Thereafter, multivariate logistic regression analyses (forced entry method) were utilised to assess the independent effect of variables that were statistically significant in the univariate analyses. Those coded as zero were the reference categories. The four dependent variables (recorded as yes = 1, no = 0) were social problems (homelessness or unemployment or threats of violence); infectious diseases (HIV or Hepatitis A or Hepatitis B or Hepatitis C); psychological problems (depressive symptoms or psychotic symptoms or suicidal thoughts or suicide attempt); and a combination of social and health problems (i.e. combinations of social problems, infectious diseases and psychological problems). Independent variables considered included age (in years), gender (male = 1, female = 0); marital status (married = 1, unmarried = 0); education (≥ high school = 1, < high school = 0); children under 18 years (yes = 1, no = 0); referral to treatment (self-referral = 1, other referrals = 0); use of alcohol as primary drugs (yes =1, no = 0); use of cannabis as primary drugs (yes = 1, no = 0); use of prescription medicines as primary drugs (yes = 1, no = 0); use of opiates as primary drugs (yes = 1, no = 0); use of stimulants as primary drugs (yes =1, no = 0); use of other drugs as primary drugs (yes = 1, no = 0). Other primary drugs refer to hallucinogens, solvents/inhalants, gamma-hydroxybutyric acid and anabolic steroids. Results were expressed as adjusted odds ratios (AORs) with 95% confidence intervals (CIs). All analyses were carried out using SPSS version 17 for windows (SPSS Inc., Chicago IL).

## Results

### Seeking treatment

Substance use was the main complaint (89%, *n* = 2237). Other main concerns for seeking treatment were related to social (4%, n = 91), psychiatric (1%, *n* = 32), physical (0.1%, *n* = 2), aftercare (0.1%, *n* = 2) and other unspecified issues (3%, *n* = 73). Half of the respondents (50%, *n* = 1258; Table 1) sought treatment on their own. Non-voluntary referrals were by family and friends (19%, *n* = 489); the police (1%, *n* = 14); employers (0.3%, *n* = 7); child healthcare (4%, *n* = 92); and others including healthcare, school and social services. Thirty-two percent (*n* = 811) had previous contact with drug treatment. In addition to their current treatment, about 25% (*n* = 622) were receiving treatment elsewhere (Table 1).

**Table 1:** Background characteristics of clients at first visit to HDI, 2001 – 2008

**Variable**	**N = 2526**
**Age**	
Mean age (SD)	25 (±8.4)
Missing data	-
**Gender, *n (%)***	
Male	1702 (67)
Female	824 (33)
Missing data	-
**Marital status, *n (%)***	
Married or cohabiting	197 (8)
Unmarried ^a^	1869 (74)
Missing data	460 (18)
**Education **^**b**^**, *n (%)***	
≥ High school	589 (23)
< High school	1766 (70)
Missing data	171 (7)
**Children under 18 years, *n (%)***	
Yes ^c^	599 (24)
No	1713 (68)
Missing data	214 (8)
**Referral to treatment**, ***n (%)***	
Self-referral	1258 (50)
Other referrals ^d^	1184 (47)
Missing data	84 (3)
**Primary drugs of abuse, *n (%)***	
Use of alcohol as primary drugs	659 (26)
Use of cannabis as primary drugs	382 (15)
Use of prescription medicines as primary drugs	55 (2)
Use of opiates as primary drugs	788 (31)
Use of stimulants as primary drugs	599 (24)
Use of Other drugs as primary drugs ^e^	43 (2)
Missing data	-

HDI – Helsinki Deaconess Institute.

^a^ including – unmarried, separated/divorced or widowed.

^b^ < high school = elementary school, ≥ high school = high/vocational school, university and others.

^c^ Including those children living in the same household, in foster care, elsewhere or unspecified.

^d^ Referral by friends and family, the police, employers and other sources.

^e^ Others – including hallucinogens, solvents/inhalants, gamma-hydroxybutyric acid and anabolic steroids.

### Demographic and living condition variables

As shown in Table 1, the mean age of the clients was 25 years (standard deviation [SD] ±8.4). Seventy percent (*n* = 1766) were educated below high school level. Less than ten percent were married and others were either unmarried (68%, *n* = 1707), separated/divorced (6%, *n* = 161) or widowed (0.04%, *n* = 1). About two-thirds (68%, *n* = 1713) reported that they did not have children less than 18 years. However, others had children less than 18 years living in the same household (6%, *n* = 159), in foster care (4%, *n* = 110), elsewhere (12%, *n* = 296), or unspecified places (1%, *n* = 34). Forty-four percent (*n* = 1110) did not have other people who use drugs living with them. Among those who did, other individuals reported to be living in the same household used illicit drugs (14%, *n* = 345), alcohol (3%, *n* = 62) or both illicit drugs and alcohol (2%, *n* = 50).

### Drug use patterns

Details of drug use patterns among all the clients have been described elsewhere [27] but a brief description is provided here. Lifetime prevalence was highest for cannabis (78%). Almost 40% were using five drugs at the time of interview, signifying polydrug use. Opiates were the most frequently reported primary drug of abuse (30%). Forty-five percent administered their primary drugs intravenously and 44% used their primary drugs daily during the past month. Cannabis was the most common secondary drug (34%). The secondary drugs were mostly smoked (39%) and were mostly consumed once per week or less frequently (33%) during the past month.

### Social problems reported at first visit

About one-fifth of the clients (21%, *n* = 519) did not have postal codes/addresses and were considered to be homeless (Table 2). Fifty-four percent (*n* = 1363) were not employed at the time of seeking treatment. For 18% (*n* = 446), their salaries were their main sources of income. Other clients reported that their main income was from pension (3%, *n* = 77), income support (32%, *n* = 811), unemployment benefit (12%, *n* = 294) and other unspecified sources (14%, *n* = 353). Threats of violence were reported by 7% (*n* = 183). Sociodemographic and drug use characteristics associated with social problems in univariate and multivariate logistic models were shown in Tables 3 and 4 respectively. Those who reported social problems were significantly more likely than other clients to be self-referred to treatment (AOR = 1.86; 95% confidence interval (CI) = 1.50–2.30]; to be male (AOR = 1.48; 95% CI = 1.19–1.84); to be older (AOR = 1.04; 95% CI = 1.03–1.06); not using alcohol (AOR = 0.49; 95% CI = 0.29–0.83) and cannabis (AOR = 0.47; 95% CI = 0.27–0.82) as primary drugs of abuse.

**Table 2:** Social and health problems reported by the clients, 2001–2008 (N = 2526)

**Variable**	**Yes**	**No**	**Missing data**	**Total**
	**n (%)**	**n (%)**	**n (%)**	**n (%)**
**1. Social problems**				
Homelessness	519 (21)	1971 (78)	36 (1)	2526 (100)
Unemployment ^a^	1363 (54)	1030 (41)	133 (5)	2526 (100)
Threats of violence	183 (7)	1819 (72)	524 (21)	2526 (100)
**2. Infectious disease screening tests**				
HIV positive	48 (2)	1383 (55)	1095 (43)	2526 (100)
Hepatitis A positive	70 (3)	1219 (48)	1237 (49)	2526 (100)
Hepatitis B positive	84 (3)	1241 (49)	1201 (48)	2526 (100)
Hepatitis C positive	630 (25)	763 (30)	1133 (45)	2526 (100)
**3. Psychological problems**				
Depressive symptoms	1490 (59)	726 (29)	310 (12)	2526 (100)
Psychotic symptoms	430 (17)	1761 (70)	335 (13)	2526 (100)
Suicidal thoughts	745 (30)	1458 (58)	323 (12)	2526 (100)
Suicide attempts	478 (19)	1579 (63)	469 (18)	2526 (100)

^a^No – includes employed persons, students, retirees, housewives/househusbands.

**Table 3:** Univariate logistic regression analyses of factors associated with social problems, infectious diseases, psychological problems and all three of these outcomes (N = 2526)

**Characteristics**	**Social problems**^**1**^	**Infectious diseases**^**2**^	**Psychological problems**^**3**^	**Social problems**^**1**^**, infectious diseases**^**2**^**, and psychological problems**^**3**^
	**Unadjusted OR (95% CI)**	**Unadjusted OR (95% CI)**	**Unadjusted OR (95% CI)**	**Unadjusted OR (95% CI)**
Age (in years)	1.07 (1.06–1.09)*	1.10 (1.09–1.11)*	1.06 (1.05–1.07)*	1.07 (1.06–1.08)*
Male gender	1.50 (1.27–1.77)*	1.26 (1.04–1.52)*	0.75 (0.62–0.90)*	1.23 (0.98–1.55)
Married or cohabiting	1.46 (1.07–1.99)*	1.76 (1.29–2.41)*	1.22 (0.89–1.68)	1.31 (0.90–1.91)
≥ High school education	0.97 (0.80–1.18)	0.98 (0.79–1.21)	1.58 (1.27–1.95)*	0.89 (0.69–1.14)
Having children under 18 years	1.95 (1.59–2.39)*	2.74 (2.25–3.33)*	1.57 (1.26–1.94)*	2.05 (1.64–2.58)*
Self-referral to treatment	2.82 (2.38–3.34)*	2.17 (1.80–2.61)*	2.08 (1.75–2.48)*	2.19 (1.75–2.73)*
Use of alcohol as primary drugs^a^	0.39 (0.32–0.46)*	0.48 (0.38–0.60)*	0.54 (0.45– 0.65)*	0.51 (0.39–0.67)*
Use of cannabis as primary drugs^a^	0.38 (0.31–0.48)*	0.16 (0.10–0.24)*	0.51 (0.41–0.63)*	0.18 (0.10–0.30)*
Use of prescription medicines as primary drugs^a^	1.05 (0.60–1.81)	0.47 (0.22–0.99)*	1.21 (0.67 – 2.18)	0.73 (0.33–1.62)
Use of opiates as primary drugs^a^	3.03 (2.50–3.68)*	2.60 (2.17–3.13)*	1.72 (1.42–2.07)*	2.45 (1.97–3.03)*
Use of stimulants as primary drugs^a^	1.72 (1.41–2.10)*	1.62 (1.33–1.98)*	1.82 (1.47–2.24)*	1.40 (1.11–1.77)*
Use of other drugs as primary drugs^a^	0.99 (0.53– 1.83)	0.45 (0.19–1.06)	1.03 (0.54–1.95)	0.37 (0.12–1.21)

* P ≤ 0.05. ^a^ Other drugs – including hallucinogens, solvents/inhalants, gamma-hydroxybutyric acid and anabolic steroids. OR – odds ratio. CI – confidence interval. ^1^Homelessness or unemployment or threats of violence. ^2^HIV or hepatitis A or hepatitis B or hepatitis C. ^3^Depressive symptoms or psychological symptoms or suicidal thoughts or suicide attempts.

**Table 4:** Multivariate logistic regression analyses of factors associated with social conditions, infectious diseases, psychological problems and all three of these outcomes (N = 2526)

**Characteristics**	**Social problems**^**1**^	**Infectious diseases**^**2**^	**Psychological problems**^**3**^	**Social problems**^**1**^**, infectious diseases**^**2**^**, and psychological problems**^**3**^
	**Adjusted OR (95% CI)**	**Adjusted OR (95% CI)**	**Adjusted OR (95% CI)**	**Adjusted OR (95% CI)**
Age (in years)	1.04 (1.03–1.06)*	1.08 (1.06–1.10)*	1.06 (1.04–1.08)*	1.06 (1.04–1.07)*
Male gender	1.48 (1.19–1.84)*	1.03 (0.80–1.31)	0.65 (0.52–0.80)*	-
Married or cohabiting	0.93 (0.65–1.33)	0.99 (0.70–1.41)	-	-
≥ High school education	-	-	1.04 (0.81–1.32)	-
Having children under 18 years	1.14 (0.86–1.53)	1.40 (1.06–1.84)*	0.86 (0.65–1.12)	1.21 (0.93–1.57)
Self-referral to treatment	1.86 (1.50–2.30)*	1.31 (1.03–1.67)*	1.51 (1.23–1.85)*	1.36 (1.07–1.74)*
Use of alcohol as primary drugs	0.49 (0.29–0.83)*	1.48 (0.49–4.45)	0.66 (0.39–1.12)	1.40 (0.66–2.95)
Use of cannabis as primary drugs	0.47 (0.27–0.82)*	0.55 (0.17–1.82)	0.68 (0.39–1.19)	0.49 (0.20–1.21)
Use of prescription medicines as primary drugs	-	0.62 (0.15–2.63)	-	-
Use of opiates as primary drugs	1.67 (0.99–2.83)	3.89 (1.32–11.46)*	1.11 (0.65–1.90)	3.24 (1.58–6.65)*
Use of stimulants as primary drugs	1.23 (0.72–2.08)	2.76 (0.92–8.20)	1.29 (0.75–2.23)	2.28 (1.10–4.72)*
Use of other drugs as primary drugs ^a^	-	-	-	-

* P ≤ 0.05. ^a^ Others drugs – including hallucinogens, solvents/inhalants, gamma-hydroxybutyric acid and anabolic steroids. Only variables statistically significant in univariate analyses were included in multivariate analyses. OR – odds ratio. CI – confidence interval. ^1^Homelessness or unemployment or threats of violence. ^2^HIV or hepatitis A or hepatitis B or hepatitis C. ^3^Depressive symptoms or psychological symptoms or suicidal thoughts or suicide attempts.

### Health problems reported at first visit

#### Infectious diseases

HIV was the least reported infectious disease and the prevalence of positive screening tests was 2% (*n* = 48; Table 2). The prevalence of positive tests for other infectious diseases included 3% (*n* = 70) for Hepatitis A, 3% (*n* = 84) for Hepatitis B and 25% (*n* = 630) for Hepatitis C. Based on the route of administration of the primary drugs, reports of a positive screening test for HIV (71%), Hepatitis A (79%), Hepatitis B (74%) and for Hepatitis C (74%) were higher among intravenous drug users than the others. As shown in Table 4, factors associated with reporting positive screening test for infectious diseases included using opiates as primary drugs of abuse (AOR = 3.89; 95% CI = 1.32–11.46); having children under 18 years (AOR = 1.40; 95% CI = 1.06–1.84); being self-referred to treatment (AOR = 1.31; 95% CI = 1.03–1.67); and being older (AOR = 1.08; 95% CI = 1.06–1.10).

#### Psychological problems

Overall, depressive symptoms were the most prevalent psychological problems (59%, *n* = 1490; Table 2). Seventeen percent (*n* = 430) reported psychotic symptoms at the time of interview. Thirty percent (*n* = 745) reported having suicidal thoughts and almost one-fifth (19%, *n* = 478) of the respondents have attempted suicide. As shown in Table 4, clients who reported psychological problems were significantly more likely than others to be self-referred to treatment (AOR = 1.51; 95% CI **=** 1.23–1.85); to be older (AOR = 1.06; 95% CI = 1.04–1.08); and not to be male (AOR = 0.65; 95% CI = 0.52–0.80).

Further analyses were carried out on those clients who reported a combination of social and health problems. Multivariate analysis (Table 4) showed that factors associated with reporting both social and health problems included using opiates (AOR = 3.24; 95% CI = 1.58–6.65) and using stimulants (AOR = 2.28; 95% CI = 1.10–4.72) as primary drugs of abuse; being self-referred to treatment (AOR = 1.36; 95% CI = 1.07–1.74); and being older (AOR = 1.06; 95% CI = 1.04–1.07).

## Discussion

This study reports baseline data on social conditions and health problems from the first large-scale longitudinal study of treatment-seeking illicit drug users in Finland. Findings from this study highlighted some level of housing instability among treatment-seekers. This lends credence to results from research studies in other countries, which found cases of homelessness and street-dwelling among those who use drugs [28-30]. Galea and Vlahov [31] argue that homelessness is an important social circumstance that influences the health and well-being of drug users. For example, homeless drug users engage in risky behaviours such as trading sex for drugs and money. IDUs who are homeless may consume drugs in public unhygienic environments, which increase the risk of infection at injection sites and sharing of injecting equipment [32]. In our study, few clients received threats of violence; this differed from the results of a large-scale study conducted in Canada [33], where a large proportion of illicit drug users reported actual experiences of violence. Therefore, our finding might suggest limited hostility from both drug-using and non-drug using people in Finland.

Unemployment was common. There was a disparity between the number of people who were employed and those who reported salary as their main sources of income; it could be that some clients who were not officially classified as being employed (e.g. students) also earned salary through part-time work. However, collectively, nearly half of the respondents were dependent on government funding in terms of income support and unemployment benefits. Given their low economic capabilities, it is likely that many clients will continue to depend on public funding for sustenance. Therefore, drug-using clients could mount financial pressure on social welfare services.

Some clients reported living within the same household with other legal and illicit drug users. Association with other drug users helps to sustain drug-using habits [34] and may hamper success in the treatment programme. One hundred and fifty-nine clients (6%) reported having children less than 18 years living in the same household; this might lead to early exposure and transfer of drug-using habits to children. Previous studies have demonstrated that children living in such social environments are predisposed to drug use in their adulthood [35,36]. Since the likelihood of reporting infectious diseases was 1.40 times higher among those with children less than 18 years, they might have sought treatment out of fear of losing their children or might have been referred by child care and social services.

Treatment-seeking was voluntary (self-referral) for half of the respondents. One possible explanation is that organisation and delivery of drug treatment services in Finland take place at municipality level, so that services are brought closer to drug users. Since self-referral was a significant predictor of reporting social and health problems, these concerns may have necessitated treatment seeking. Referral from the criminal justice system was one percent, which was lower than 37% reported among treatment samples in USA [37]. This might reflect differences in national drug policies, with strong emphasis on criminal justice interventions related to drug use in USA. These findings have implications for treatment outcomes. Health and other concerns provide transient motivation to clients and some may not complete treatment [38]. A British study found high drop out rates among clients coerced into treatment through the criminal justice system [39]. However, a US report [40] highlighted criminal justice referral as one of the strongest predictors of outpatient treatment completion, probably due to sanctions for non-completion.

A quarter of the respondents (25%) were receiving concurrent treatment elsewhere. This may be related to the fact that the HDI also serves as a treatment needs assessment centre, which receives referrals from other smaller clinics. However, treatment providers should aim to prevent ‘treatment shopping’ among clients. Receiving treatment from multiple centres may interfere with clients’ ability to commit to a specific treatment plan and would result in wastage of public funds. In addition, clients receiving treatment from multiple centres could receive double dose of prescription medication, which might increase the risk of excessive consumption and overdose or sales in illegal street market.

Hepatitis C was the most prevalent infectious disease and may be connected to high use of intravenous drugs in this study population. This finding is consistent with the report of Aceijas and Rhodes [41], which identified high prevalence of hepatitis C infections among IDUs in most of the 57 countries reviewed. The reported HIV seroprevalence in this study was lower than that reported elsewhere in Europe [42], possibly due to low prevalence in the general Finnish population which is currently 0.1% [43]. Using opiates as primary drugs of abuse was the strongest factor associated with reporting positive tests for infectious diseases, probably due to sharing of contaminated injecting equipment. This calls for heightened awareness of the needle exchange programmes in Finland.

Depressive symptoms were the most common psychological problems. Our result is consistent with previous research, which suggested that depressive symptoms are common among drug users [44]. Suicidal thoughts and suicide attempts reported by clients may be related to depressive symptoms, which were highly prevalent in this study population. Male clients were 0.65 times less likely to report psychological symptoms, consistent with a previous study [45], which also reported higher prevalence among female drug users. Therefore, it is important to identify clients who would benefit from a combination of mental health and drug abuse treatment in order to prevent premature deaths from suicide.

Findings from this study have implications for the publicly funded healthcare system in Finland. The existence of drug use with other social and health problems could lead to high healthcare expenditure. Low socioeconomic status, as evidenced by unemployment, low education level, and homelessness, influence risk-taking behaviours and contribute to negative health consequences among those who use drugs [31]. Therefore, there is a need to get drug users into early treatment in order to reduce the financial costs of care. A study conducted among drug users entering treatment in the US found a decline in total medical cost in the post-treatment period [46]. Co-occurring psychological and medical problems should be addressed to prevent relapse and excessive utilisation of health services [19].

### Limitations of the study

Incomplete information for some variables, especially the infectious diseases, suggests that the clinical staff may not have recorded some of the clients’ responses during the initial interview. For example, some clients may have answered no to some of the questions but this was not explicitly recorded. The presence of missing data highlights some of the challenges encountered when using clinical data for research purposes. Improvements in the completeness of medical data will enhance its utility for research purposes. A report from the UK argues that improvements in the standard of medical documentation are essential for planning services and for efficient patient care [47].

Our study relied on self-reported data, the veracity of which cannot be assured. However, a study by Kokkevi and colleagues [24] found a high reliability of self-reported information by drug users. Previous research in the UK suggests that drug users are not unwilling to discuss stigmatised behaviours such as sharing of injecting equipment with researchers [48]. We have no reason to assume that clients seeking treatment (largely voluntarily) were not honest in their responses in relation to their social, health and medical status, particularly when public trust in governmental/administrative institutions in Finland is reasonably high. However, it is possible that clients got their test results for HIV and Hepatitis wrong and cross-validation with laboratory investigations would have been useful. That said, the high prevalence of hepatitis C infection in this study was comparable to other European studies among illicit drug users validated with saliva and blood specimens [49-51]. Psychological data were not measured using validated scales, but the clinicians working within the treatment setting had the knowledge and skills required to carry out psychological assessments. Clients seeking treatment may differ in several ways from drug users who are not in contact with the treatment system and this limits the generalisability of our findings to non-treatment seekers.

## Conclusion

This study highlighted the coexistence of illicit drug use with other social and health problems. Drug use problems do not exist in isolation. It is essential to address adverse social situations and health problems that may interfere with clients’ ability to stay focused and comply with therapy. Our findings support the call for integration of relevant social, medical and mental health support services with drug treatment programmes. Diverse opinions exist regarding the increase in the overall cost of treatment due to additional expenses on support services. However, provision of such services could be more cost-effective, at least in the long run, in reducing morbidity, boosting retention rates and facilitating the recovery process.

## Competing interests

The authors declare that they have no competing interests.

## Authors’ contributions

JK and JT were the principal investigators of the HUUTI consortium research project. INO, JK, and CB designed this particular study. MP and NT participated in data collection. INO carried out the data analyses and prepared the first draft of the manuscript. All authors participated in interpreting the results and critical revision of the manuscript for important intellectual content. All authors read and approved the final manuscript.

## Pre-publication history

The pre-publication history for this paper can be accessed here:

http://www.biomedcentral.com/1471-2458/13/380/prepub
